# A Comparative Study of Demographic and Clinical Criteria Between Male and Female Patients With Pemphigus Referred to a Referral Hospital in Iran

**DOI:** 10.1155/2024/9572303

**Published:** 2024-10-24

**Authors:** Zeinab Aryanian, Kamran Balighi, Nafiseh Esmaeli, Maryam Daneshpazhooh, Nasim Mazloomi Tootoonchi, Zahra Razavi, Fereshteh Beigmohammadi, Umamah Gul, Azadeh Khayyat, Parvaneh Hatami

**Affiliations:** ^1^Autoimmune Bullous Diseases Research Center, Tehran University of Medical Sciences, Zip Code 1199663911, Tehran, Iran; ^2^Department of Dermatology, School of Medicine Razi Hospital, Tehran University of Medical Sciences, ZIP Code 1199663911, Tehran, Iran; ^3^Department of Dermatology, Babol University of Medical Sciences, ZIP Code 4717647745, Babol, Iran; ^4^PGY2 Resident Physician, Pathology Department of Medical College of Wisconsin, Milwaukee, Wisconsin, USA

**Keywords:** cutaneous, demographic, mucosal, mucocutaneous lesions, pemphigus, severity

## Abstract

**Background:** Pemphigus is a rare autoimmune disease characterized by the formation of blisters on the skin and mucous membranes, caused by autoantibodies against desmoglein, a key protein in cell adhesion. This study aims to compare demographic and clinical criteria between male and female patients with pemphigus referred to a referral hospital, utilizing data from the pemphigus diseases registry.

**Method:** This retrospective cross-sectional analysis focused on several key aspects age at disease onset, severity (measured by the Pemphigus Disease Area Index [PDAI]), types of pemphigus, duration of disease, and diagnostic criteria including the presence of antidesmoglein antibodies and findings from direct immunofluorescence (DIF). By examining these variables among a cohort selected based on their diagnosis of pemphigus, the study aimed to identify significant gender differences in disease manifestation, diagnosis, and progression. This approach is crucial for tailoring more effective gender-specific management and treatment strategies for this rare autoimmune condition.

**Results:** In a comprehensive analysis of 1218 pemphigus patients in the year 2021 from the hospital's registry, comprising 543 males (44.6%) and 675 females (55.4%), significant gender differences were identified in 9 out of 44 variables examined. The study revealed that males had a higher age at disease onset, more frequent clinical manifestations in the head, neck, and trunk areas, and greater severity of disease as measured by the PDAI score compared to females. Conversely, females exhibited higher instances of mucosal manifestations and a higher PDAI score for mucosal erosion blister of the lower gingiva. No significant gender differences were found in 21 variables, including the overall age of patients, specific clinical manifestations across various mucous membranes, types of pemphigus, and PDAI scores for mucosal erosions in particular locations, indicating a nuanced gender impact on the presentation and severity of pemphigus that necessitates tailored clinical approaches.

**Conclusion:** The study highlights significant gender differences in the presentation and severity of pemphigus, underscoring the importance of gender-specific approaches in the diagnosis and management of this condition. The findings contribute valuable insights into the complex nature of pemphigus and underline the necessity for further research to understand the underlying mechanisms driving these differences.

## 1. Introduction

Pemphigus encompasses a range of rare autoimmune blistering diseases that significantly impact the skin and mucous membranes, leading to considerable morbidity and, in some instances, mortality among affected populations. This group of disorders, termed by the existence of autoantibodies against desmogleins, results in the loss of cell adhesion within the epidermis, a hallmark called acantholysis. Laboratory reference ranges for antidesmoglein antibodies (Dsg1 and Dsg3) typically classify results as negative if less than 20 U/mL, indeterminate or borderline if lower between 20 and 29 U/mL, and positive if it is 30 U/mL and positive if it is 30 U/mL or more. These locations are standard for ELISA-based tests for the diagnosis of pemphigus vulgaris and pemphigus foliaceus, although there may be slight variation depending on the specific test and laboratory equipment used. The pathophysiological cascade underpins the clinical symptoms of the pemphigus, which range from the presence of blisters and sores across various areas of the body [1, 2].

Studies have highlighted associations with other autoimmune conditions, certain drugs, and specific demographic groups, suggesting factors affecting disease onset and outcome [3–6]. The epidemiology of pemphigus is characterized by variability, with its prevalence being affected by geographic locations and demographic characteristics of the population. A gender effect has been documented in the occurrence of pemphigus, with a higher proportion reported among females in various studies [3, 7–10].

This study aims to conduct a comprehensive comparative analysis of demographic and clinical criteria between male and female patients with pemphigus referred to a referral hospital. By leveraging data recorded in the Pemphigus Diseases Registry, this research seeks to elucidate potential gender-based differences in disease manifestation, course, and response to treatment. Through this investigation, we aspire to contribute valuable insights into the nuanced nature of pemphigus, facilitating more tailored and effective approaches to the management of this challenging group of diseases.

## 2. Method

In this retrospective cross-sectional study, data were sourced from the comprehensive pemphigus disease registry, focusing on a cohort of 2000 patients in the year 2021 to conduct a comparative analysis of demographic and clinical criteria between male and female patients diagnosed with pemphigus. The investigation spanned a broad spectrum of independent variables, including demographic details, clinical presentations, autoimmune disease associations, and specific pemphigus-related criteria such as antidesmoglein antibody presence, Pemphigus Disease Area Index (PDAI) scores, and direct immunofluorescence (DIF) testing results. Inclusion criteria included the diagnosis of any type of pemphigus, either pemphigus vulgaris or pemphigus foliaceus, which was confirmed in patients through clinical examination, histopathology, and DIF examination, patients of both sexes over 18 years, and patients whose complete medical records include demographic data, clinical data, laboratory examinations, and treatment history. Patients treated with systemic corticosteroids, such as prednisolone, and/or rituximab. Exclusion criteria included patients with incomplete or missing medical records, particularly regarding key study variables such as PDAI scores or antidesmoglein antibody levels, patients under 18 years of age, patients diagnosed with other autoimmune blistering diseases that are not classified as pemphigus, and patients who were lost to follow-up before completing the treatment course or who did not receive standard treatment modalities (e.g., systemic corticosteroids or rituximab).

Dependent variables were primarily concerned with treatment modalities, particularly prednisolone dosages at various treatment stages and the number of rituximab cycles administered. Data collection entailed a detailed review of patient files and registry entries to extract relevant demographic, clinical, laboratory, and pathological information, aiming to identify gender-based differences in disease manifestation, severity, and progression.

The Shapiro–Wilk test was used in the statistical analysis to determine if the data were normal. Continuous variables were described using means and standard deviations, while categorical variables were represented using frequencies and percentages. Using descriptive statistics, Chi-square tests, Fisher's exact test for qualitative variables, independent *t*-tests, and Mann–Whitney tests for quantitative variable comparison, the significance of the findings was assessed at a 95% confidence level (*p* value < 0.05). Even though Stata software was initially mentioned as a means of data analysis, R software was eventually employed for all statistical studies, with a significance level of 0.05. The pemphigus patient population at Razi Hospital was thoroughly analyzed and thanks to this scientific approach, which also shed light on the subtle variations in treatment outcomes and clinical presentations between male and female patients.

## 3. Result

In the study, a comparative analysis of demographic and clinical criteria between male and female patients with pemphigus was conducted, utilizing data recorded in the Pemphigus Diseases Registry. The research encompassed 1218 cases, comprising 543 (44.6%) males and 675 (55.4%) females, indicating a higher percentage among females.

A notable finding was the significant difference in smoking habits between genders (*p*=0.018), with smoking being more frequent among males.

Age at disease onset varied between males (mean age of onset 46 years) and females (mean age of onset 42 years), suggesting earlier diagnosis manifestation in males (*p*=0.02). Duration of the disease also revealed a gender difference (*p*=0.008), with males developing a shorter duration compared to females (5.14 months vs. 7.30 months) (Table 1).

Clinically, male patients showed a higher frequency of head and neck area involvement (30.76%) compared to females (17.63%) and trunk skin involvement (11.05% in males vs. 5.93% in females), indicating gender-specific disease manifestation patterns.

The PDAI score for mucosal erosion blister of lower gingiva was significantly higher in females (mean = 0.36) than in males (mean = 0.17), pointing toward a more severe mucosal disease manifestation in females (*p* < 0.001) (Table 1).

The incidence of mucocutaneous lesions varied significantly at the time of diagnosis, with men experiencing a higher frequency (31.68%) than women (19.26%). The likelihood of mucosal lesions at the time of diagnosis is observed to be 28.59% in females and 16.57% in males. Additionally, the occurrence of cutaneous lesions is recorded at 16.76% in males and 12.15% in females (Table 2).

Men had a higher mean severity rating (4.6) than females (3.1) based on severity ratings, suggesting that men had a more severe disease course (*p*=0.011) (Table 3).

We analyzed 44 different variables to compare data between genders. However, some variables had missing data, making the conclusions drawn from those variables less reliable. The variables with complete data provided more robust conclusions, which are discussed and compared to previous studies on the subject. No conclusions were made for variables with missing data, including current address, ethnicity, birthplace type, smoking habits, alcohol use, illicit drug abuse, presence of stressors, anti-Dsg1 and anti-Dsg3 levels, disease duration, and past medical history.

## 4. Discussion

In this study, we aimed to investigate the demographic and clinical differences of the pemphigus registry in Razi Dermatology Hospital in Tehran between sexes. In this data pool, females were slightly prevalent (55.4%) over males. The difference was minimal. In that case, the gender distribution is consistent with global findings that were reported by Marazza et al., in that a high incidence of pemphigus was also found in women. This suggests that pemphigus can affect both men and women, but females may have a slight but consistent prevalence throughout the world [4].

An important observation was sex-based significant difference in smoking *p*=0.018 with males having higher smoking rates. Smoking was described as a risk factor for some autoimmune diseases, including pemphigus. This is in line with results from the study by Brenner et al. on the role of cigarettes smoking as an environmental trigger in pemphigus, especially in male patients.

The higher smoking prevalence among males in our study could partially explain the more severe disease manifestations observed in this group, as smoking has been associated with increased disease activity and poorer prognosis in autoimmune conditions [8].

The age at which the first diagnosis of disease was made was also significantly different in males compared to females at 42 and 46, respectively (*p*=0.02). This is contrast to the previous study by Timoteo et al., which found no gender predominance in the disease [9].

The early male onset observed in our study may reflect regional genetic or environmental factors predisposing males to early pemphigus presentation in the Iranian population. The reduced duration of the disease in men, averaging 5.14 months, in contrast to 7.30 months in women (*p*=0.008), lends additional credence to the notion that the disease may be more severe in men, indicating that active treatment intervention may be necessary sooner.

Gender differences in clinical presentation were also evident, with males more frequently experiencing head and neck involvement (30.76% vs. 17.63% in females) and tail skin involvement (11.05% vs. 5.93% in females). These findings are consistent with studies by Barcelos et al., who reported a similar distribution pattern of the disease, with males more frequently exhibiting skin exposure revealed [5]. The higher incidence of mucocutaneous lesions at diagnosis in males (31.68%) compared to females (19.26%) highlights the need for increased medical vigilance in male patients based on their more severe disease.

Interestingly, despite greater cutaneous involvement in men, women showed more severe psoriasis, as indicated by higher PDAI scores in low-grade gingival psoriasis (average score 0.36 in females vs. 0.17 in males, *p* < 0.001). This finding is consistent with the work of Ahmed and Sami, who observed that, although males are more likely to present with severe skin reactions, females often develop severe mucosal disease [7]. This difference in disease presentation highlights the importance of gender-specific considerations in the management of pemphigus, with tailored treatment strategies that should dealt with emphasizes disease that manifests primarily in each sex.

In this study, the total disease severity measured with severity was higher in males with a mean score of 4.6 compared to females with a mean score of 3.1 with *p*=0.011. This result is similar to the data provided by Thomas et al., who has observed more severe course of the disease in male patients, especially in ocular involvement [6]. This suggests that, as compared to women, men were more severe in our study and may need more aggressive treatment strategies, including early initiation of rituximab-like therapy. Balighi et al. showed better outcome with early use of rituximab [1].

In our study, some variables, such as anti-Dsg1 and anti-Dsg3 levels, duration of disease, and past medical history, had missing data. These differences in data collection seriously limited the possibility of completing full data in all of these areas. Variables, however, still turn out relevant in answering the research question of gender differences in pemphigus and furthering the answer to these differences by implying more research on mechanisms underlying these differences.

This research adds to the currently accumulating evidence on the key sex-based differences in the clinical presentation and severity of pemphigus. The findings reiterate that sex is an important variable in diagnosis, management, and prognosis among patients with pemphigus. In this way, further research should aim to provide clarification regarding genetic, biochemical, and environmental factors of predisposing differences that could be helpful in developing more individual and effective treatment strategies for patients with pemphigus.

## Figures and Tables

**Table 1:** Age of onset, duration of disease, and PDAI score for mucosal erosion blister of lower gingiva and scalp regarding gender.

Age at disease onset									
Gender	*N*	Mean	SD	Min	Q1	Median	Q3	Max	*p* value
Male	116	46.74	13.64	19	37	45.5	56	86	0.02
Female	137	42.59	14.43	13	32	41.0	51	82	

**Duration of the disease**									
**Gender**	***N***	**Mean**	**SD**	**Min**	**Q1**	**Median**	**Q3**	**Max**	**p** **value**

Male	116	5.14	4.00	Less than a year	2	3.5	8	18	0.008
Female	137	7.30	6.72	Less than a year	3	5.0	9	35	

**PDAI score for mucosal erosion blister of lower gingiva**									
**Gender**	***N***	**Mean**	**SD**	**Min**	**Q1**	**Median**	**Q3**	**Max**	**p** **value**

Male	543	0.17	0.74	0	0	0	0	10	<0.001
Female	675	0.36	1.13	0	0	0	0	10	

**PDAI score for mucosal erosion blister of scalp**									
**Gender**	***N***	**Mean**	**SD**	**Min**	**Q1**	**Median**	**Q3**	**Max**	**p** **value**

Male	543	0.64	1.67	0	0	0	1	10	<0.001
Female	675	0.26	1.15	0	0	0	0	10	

**Table 2:** The frequency of mucosal, cutaneous, and mucocutaneous clinical manifestations at the time of diagnosis.

Gender	Mucosal	*N*	Percent	*p* value
Male	Negative	453	83.43	<0.001
	Positive	90	16.57	
Female	Negative	482	71.41	
	Positive	193	28.59	

**Gender**	**Cutaneous**	***N***	**Percent**	**p** **value**

MALE	Negative	452	83.24	0.027
	Positive	91	16.76	
Female	Negative	593	87.85	
	Positive	82	12.15	

**Gender**	**Mucocutaneous**	***N***	**Percent**	**p** **value**

Male	Negative	371	68.32	<0.001
	Positive	172	31.68	
Female	Negative	545	80.74	
	Positive	130	19.26	

**Table 3:** Gender and disease severity, DSG1, and DSG3 in both genders.

Gender	*N*	Mean	SD	Min	Q1	Median	Q3	Max	*p* value
*Gender and severity of disease*									
Male	543	4.683	8.644	0	0	1	6	77	0.011
Female	675	3.147	6.479	0	0	0	3	51	

*DSG1 in males and females*									
Male	118	76.70	83.13	0	4.25	26.65	157.25	250	0.002
Female	161	53.11	80.35	0	1.60	5.30	104.00	250	

*DSG3 in males and females*									
Male	101	118.93	116.11	0.0	11.8	115	200	845	0.033
Female	157	88.76	86.95	0.5	4.9	51	191	250	

## Data Availability

The data that support the findings of this study are available from the corresponding author upon reasonable request.
